# Association of circulating B-type natriuretic peptide with osteoporosis in a Chinese type 2 diabetic population

**DOI:** 10.1186/s12891-021-04138-3

**Published:** 2021-03-10

**Authors:** Pan Chen, Pijun Yan, Qin Wan, Zhihong Zhang, Yong Xu, Ying Miao, Jun Yang

**Affiliations:** 1 Department of Endocrinology, The Affiliated Hospital of Southwest Medical University, Luzhou, 646000 Sichuan China; 2 Department of General Medicine, The Affiliated Hospital of Southwest Medical University, No. 25 Taiping street, Luzhou, 646000 Sichuan China

**Keywords:** B-type natriuretic peptide, Diabetic osteoporosis, Inflammation, Vasculopathy, Cardio-metabolic risk factors

## Abstract

**Background:**

Altered circulating levels and genetic variation of B-type natriuretic peptide (BNP), has been associated with lower bone mineral density (BMD) values and incidence of osteoporosis in peritoneal dialysis patients, renal transplant recipients, and postmenopausal women. The potential relationship of circulating BNP with osteoporosis in patients with type 2 diabetes mellitus (T2DM), however, has not yet been studied.

**Methods:**

Circulating BNP levels were measured in 314 patients with T2DM, and participants were divided into normal BMD group (*n* = 73), osteopenia group (*n* = 120), and osteoporosis group (*n* = 121). The association of circulating BNP with diabetic osteoporosis and other parameters was analyzed.

**Results:**

Circulating BNP was significantly higher in diabetic osteoporosis subjects than normal and osteopenia groups (*P* < 0.01 or *P* < 0.05). Circulating BNP levels correlated significantly and positively with neutrophil to lymphocyte ratio, systolic blood pressure, urinary albumin-to-creatinine ratio, and prevalence of hypertension, peripheral arterial disease, diabetic retinopathy, peripheral neuropathy, and nephropathy, and negatively with triglyceride, fasting blood glucose, lymphocyte count, hemoglobin, estimated glomerular filtration rate, bilirubin, osteoporosis self-assessment tool for Asians, BMD at different skeletal sites and corresponding T scores (*P* < 0.01 or *P* < 0.05). After multivariate adjustment, circulating BNP remained independently significantly associated with the presence of osteoporosis (odds ratio, 2.710; 95% confidence interval, 1.690–4.344; *P* < 0.01). BMD at the femoral neck and total hip and corresponding T scores were progressively decreased, whereas the prevalence of osteoporosis was progressively increased with increasing BNP quartiles (*P* for trend< 0.01). Moreover, receiver-operating characteristic analysis revealed that the optimal cutoff point of circulating BNP to indicate diabetic osteoporosis was 16.35 pg/ml.

**Conclusions:**

Circulating BNP level may be associated with the development of osteoporosis, and may be a potential biomarker for diabetic osteoporosis.

## Background

Type 2 diabetes mellitus (T2DM), a chronic metabolic disease characterized by insulin resistance and elevated blood glucose level, impairs multiple organs and functions, which subsequently leads to microvascular diseases and macrovascularcomplications [1]. Recent evidence shows that osteoporosis has been recognized as another significant skeletal complication of diabetes [2]. It is estimated that > 200 million people have osteoporosis worldwide, posing great public health and economic burdens on society. Bone mineral density (BMD), an indication of bone mass and mineralization, is recognized as one of the major determinants of bone strength and a major tool to detect osteoporosis and predict fracture risk in the general population [3]. Interestingly, epidemiological studies have found that although BMD values are reduced, normal, or increased in patients with T2DM, compared with non-diabetic patients, the risk of fracture is increased [1, 4, 5], indicating that increased risk of fractures may be due to impaired bone quality and extra-bone factors [1], however, the underlying mechanism has not been clearly determined, and there are few effective therapies for diabetic osteoporosis. Therefore, it is of great significance to find clinically suitable indicators of osteoporosis for the early prevention and treatment of diabetic osteoporosis.

Heart failure (HF), characterized by prolonged activation of the neuroendocrine system ranging from sympathoadrenal system, natriureticm peptides,renin-angiotensin-aldosterone system (RAAS) and to updated markers of osteoprotegerin [6], is also a major cardiovascular complication of diabetes mellitus. There is evidence that HF is associated with low vitamin D, accelerated bone loss and therefore osteoporosis and increased risk of fractures, particularly in the hip region [7, 8], suggest that there is a close relationship between HF and osteoporosis, and they may share common pathogenic mechanisms [7, 9]. B type natriuretic peptide (BNP) is a neurohormone produced in and secreted from the heart in response to ventricular dilatation, pressure overload and ischemic injury, and its levels rise with age and are affected by gender, renal function, comorbidity and drug therapy [10, 11]. Numerous studies showed that circulating BNP significantly increases in patients with HF, rising in line with New York Heart Association (NYHA) class, and has emerged as a reliable marker for HF and left ventricular dysfunction [12]. Collectively, these data suggested that a potential association may exist between circulating BNP and the development of osteoporosis. Indeed, BNP mediates a variety of biological effects, including diuresis/natriuresis, peripheral vasodilation, relaxing the myocardium, anti-fibrosis, anti-inflammatory, anti-atherosclerosis, promotion of lipolysis and lipid oxidation, prevention of weight loss and suppression of the renin-angiotensin system (RAS) [7, 13–15], whereas weight loss, dyslipidemia, hypertension, atherosclerosis, vasculopathy, inflammation, and activation of the RAS have been associated with accelerated bone loss and therefore osteoporosis and increased risk of fractures [9, 16, 17], suggesting that BNP has the osteoprotective properties. Consistently, it has been reported that transgenic mice overexpressing BNP presented with skeletal overgrowth [18].

However, several studies have found that circulating N-terminal pro-BNP (NT-proBNP), a biologically inactive fragment of BNP, was associated with lower BMD values and T-scores at the lumbar spine (LS) in renal transplant recipients and peritoneal dialysis patients [16, 17]. These findings regarding the association of BNP with BMD values and osteoporosis are inconsistent and controversial, and no studies, as yet, have evaluated the association of circulating BNP with diabetic osteoporosis.

Therefore, the present study was designed to compare circulating BNP, cardio-metabolic parameters, inflammatory markers, and other vascular complications in Chinese population of T2DM patients with and without osteoporosis and assess their contributions to diabetic osteoporosis in such patients.

## Methods

### Study population

A total of 314 T2DM patients aged 45–87 years in our inpatient department between August 2012 and September 2015, who completed the measurement of BMD and circulating BNP were finally enrolled in the cross-sectional study. All patients met the all inclusion criteria and the most exclusion criteria as described previously [19]. We also excluded the patients who had cardiac arrhythmias including atrial fibrillation, aortic stenosis, myocardial infarction or unstable angina within previous 3 months, uncontrolled hypertension> 180/100 mmHg, acute respiratory failure, thromboembolic disease, hematological system diseases, acute or chronic infection, autoimmune disease such as Behcet’s disease, chronic periodontitis, psoriasis, psoriatic arthritis, rheumatoid arthritis, ankylosing spondylitis, Kawasaki Disease, and synovitis, gastrointestinal system diseases, alcohol abuse, cigarette smoking (former or current), inability to ambulate, impaired cognitive function, malignancies, pregnancy or lactation.

### Clinical and biochemical measurements

All subjects completed a standard questionnaire, including diabetic duration, lifestyle habits (alcohol consumption and cigarette smoking), previous or current diseases [diabetic retinopathy (DR), nephropathy (DN), and peripheral neuropathy (DPN), peripheral arterial disease (PAD), hypertension, stroke, coronary heart disease (CHD), and other diseases] and related medications, and performed a comprehensive physical examination according to standard procedures. Body weight, height, body mass index (BMI), systolic blood pressure (SBP), diastolic blood pressure (DBP), and pulse pressure (PP) were measured as described previously [19]. Blood samples were obtained from all individuals in early morning following an overnight fasting of at least 8 h to measured fasting blood glucose (FBG), glycated hemoglobin A1C (HbA1c), lipid profiles, including total cholesterol (TC), triglyceride (TG), high density lipoprotein cholesterol (HDL-C) and low-density lipoprotein cholesterol (LDL-C), bilirubin, including total bilirubin (TBIL), direct bilirubin (DBIL) and indirect bilirubin (IBIL), serum creatinine, cystatin C, calcium, alkaline phosphatase (ALP), hemoglobin (Hb), white blood cell (WBC), neutrophil and lymphocyte counts, neutrophil to lymphocyte ratio (NLR), fibrinogen, and circulating BNP. Circulating level of BNP was quantified using an immunocheminoluminometric assay according to the manufacturer’s instruction, and its normal range was 0–100 pg/ml. Urinary microalbumin and creatinine, urinary albumin-to-creatinine ratio (ACR), estimated glomerular filtration rate (eGFR), ankle-brachial index (ABI), vibration perception thresholds (VPT), 10 g Semmes-Weinstein monofilament (SWM) evaluation, and two-field fundus photography of eyes were determined, as we described previously [19, 20]. Osteoporosis Self-Assessment Tool for Asians (OSTA) index can be calculated using the formula of (weight in kilograms–age in years) × 0.2 [21].

### BMD measurement and diagnosis of osteoporosis

The areal BMD values of the LS, femoral neck (FN) and total hip (TH) were measured by dual X-ray absorptiometry (DXA) using a GE Lunar Prodigy and were expressed as g/cm^2^, as well as in T scores (deviation from the peak BMD) [19]. Self-reported fragility fractures, including fractures of axial (ribs, lumbar and thoracic vertebrae) and peripheral bones (forearm, humerus and femur) that resulted from minimal or moderate trauma, were verified, as we described previously [19, 22]. Those patients, who had traumatic fractures and fractures occurring at sites not characteristic of bone fragility (face, skull, tibia, fibula and femoral diaphysis), were excluded from the analysis [22]. In our present study, diabetic osteoporosis was diagnosed according to T score at any of sites on the LS, FN and TH and self-reported fragility fractures [19, 23]. All participants were subsequently divided into three groups: normal group (*n* = 73), osteopenia group (*n* = 120), and osteoporosis group (*n* = 121).

### Statistical analysis

All data were first analyzed for normal distribution using the Kolmogorov-Smirnov test, and then Levene’s test is used to test the assumption of homogeneity of variance. Data are expressed as mean ± standard deviation (SD) for continuous variables or number (percentages) for categorical variables.

Differences among more than three or more groups were assessed using one-way analysis of variance (ANOVA) (continuous variables with normally distribution and homogeneity of variance), or the Kruskal-Wallis test (covariates with nonparametric distribution and/or variance uneven). Correlation analysis was used to evaluate the relationship between circulating BNP and other variables. The associations of circulating BNP and other variables with the risk of osteoporosis were performed by the univariate and multivariable logistic regression analyses. Odds ratios (OR) and 95% confidence intervals (CI) were estimated. Then, all patients were divided into four quartile groups by circulating BNP level: Q1 (circulating BNP < 9.27 pg/ml) (*n* = 78), Q2 (9.27 pg/ml ≤ circulating BNP ≤ 21.77 pg/ml) (*n* = 79), Q3 (21.77 pg/ml < circulating BNP ≤ 60.35 pg/ml) (n = 79), and Q4 (60.35 pg/ml < circulating BNP) quartile groups (*n* = 78), and parameters related to osteoporosis among four groups were compared. Last, receiver operating characteristic (ROC) curve analysis was performed to determine the optimal cut-off point of circulating BNP for the diagnosis of osteoporosis.

All analyses were conducted using the Statistical Package for Social Sciences version 20.0 (SPSS, Chicago, IL), and a two-sided *P* value of < 0.05 was considered statistically significant.

## Results

### Circulating BNP and other clinical characteristics of studied population

**Fig. 1:**
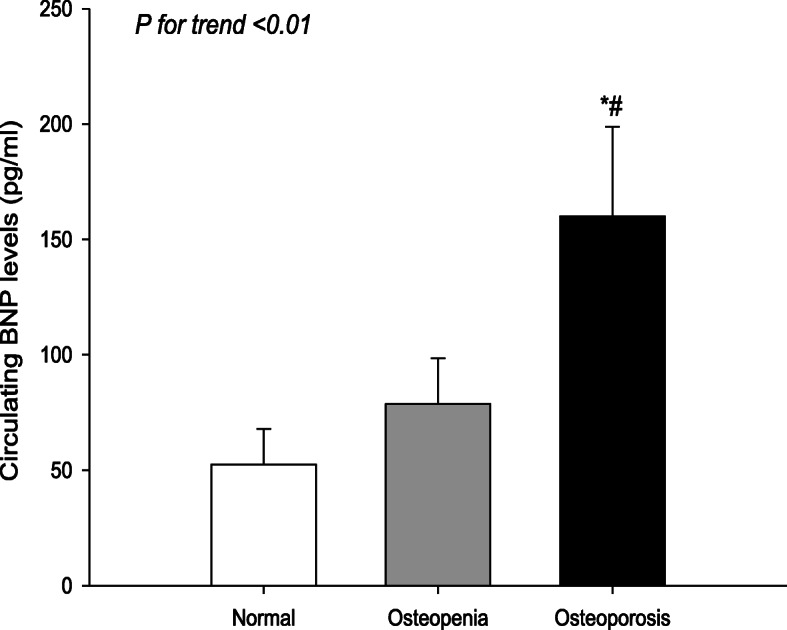
Circulating levels of BNP among three groups. Vs. Normal group, *P < 0.01; vs. Osteopenia group, ^#^*P* < 0.05

Table 1 summarizes the anthropometric, biochemical and clinical parameters of studied population. There were significant differences in anthropometric parameters (gender distribution, age, height, and weight), biomarkers of inflammation (lymphocyte count, NLR and Hb), prevalence of vascular diseases (stroke, PAD, and DN) and related indexes (PP, eGFR, ABI, and VPT), bone metabolism markers (calcium, ALP, and OSTA), BMD at different skeletal sites and corresponding T score, and prevalence of clinical fractures among three groups. When compared with those with normal BMD and osteopenia, T2DM patients with osteoporosis had significantly more female subjects, older age, larger proportions of stroke and PAD, higher NLR and BNP, and lower height, weight, lymphocyte count, Hb, ABI, calcium, OSTA, BMD at different skeletal sites and corresponding T scores (*P* < 0.01 or *P* < 0.05; Table 1 and Fig.1). They also had higher PP, VPT and prevalence of stroke and PAD, and lower eGFR, as compared to individuals with normal BMD (*P* < 0.01 or *P* < 0.05; Table 1).

**Table 1:** Circulating BNP and other clinical characteristics between T2DM patients with and without osteoporosis (*x̄*±s)

	Normal (*n* = 73)	Osteopenia (*n* = 120)	Osteoporosis (*n* = 121)	***P Value***
Male/Female	39/34	50/70	16/105**^##^	0.000
Diabetic duration (years)	7.11 ± 5.91	8.60 ± 7.19	9.43 ± 7.46	0.134
Age (years)	59.32 ± 11.98	64.12 ± 8.76*	69.49 ± 7.89**^##^	0.000
Height (cm)	161.98 ± 8.35	158.19 ± 7.75**	152.71 ± 11.83**^##^	0.000
Weight (kg)	65.86 ± 10.73	61.95 ± 9.38*	56.96 ± 13.55**^##^	0.000
BMI (kg/m^2^)	25.02 ± 3.00	24.91 ± 3.99	23.83 ± 4.33	0.058
SBP (mmHg)	132.14 ± 21.95	134.84 ± 21.80	136.71 ± 23.89	0.396
DBP (mmHg)	70.44 ± 12.01	69.73 ± 12.23	67.74 ± 13.53	0.291
PP (mmHg)	61.70 ± 19.77	65.12 ± 18.54	68.97 ± 20.65*	0.041
TC (mmol/L)	4.89 ± 109	4.81 ± 1.29	4.79 ± 1.22	0.849
TG (mmol/L)	2.31 ± 1.48	2.20 ± 1.97	1.91 ± 1.08	0.182
HDL-C (mmol/L)	1.13 ± 0.28	1.23 ± 0.40	1.25 ± 0.37	0.068
LDL-C (mmol/L)	2.89 ± 0.85	2.74 ± 1.01	2.82 ± 1.02	0.594
FBG (mmol/L)	10.40 ± 4.49	11.44 ± 6.20	10.25 ± 5.70	0.298
HbA1c (%)	9.86 ± 2.38	9.54 ± 2.62	9.12 ± 2.45	0.118
Calcium (mg/dL)^a^	9.67 ± 1.16	9.51 ± 1.09	9.21 ± 1.41**^#^	0.003
ALP (U/L)	93.56 ± 47.76	79.29 ± 39.98**	95.36 ± 46.67^##^	0.000
Creatinine (μmol/L)	77.23 ± 50.41	82.12 ± 72.08	79.75 ± 69.91	0.516
eGFR (mL/min/1.73 m^2^)	91.88 ± 31.76	85.24 ± 26.55	79.33 ± 26.66**	0.007
ACR (mg/g)	362.60 ± 151.53	132.57 ± 43.85	325.48 ± 79.67	0.143
Cystatin C (mg/L)	1.05 ± 0.48	1.12 ± 0.54	1.22 ± 0.68	0.181
WBC count (*10^9^ /L)	6.90 ± 2.06	6.88 ± 2.65	7.42 ± 3.94	0.341
Neutrophil count (*10^9^ /L)	4.68 ± 1.78	4.67 ± 2.42	5.52 ± 3.94	0.273
Lymphocyte count (*10^9^/L)	1.67 ± 0.62	1.67 ± 0.76	1.42 ± 0.56**^##^	0.006
NLR	3.26 ± 0.28	3.35 ± 0.24	4.86 ± 0.49**^##^	0.003
Fibrinogen (g/L)	3.70 ± 1.15	3.52 ± 1.17	3.98 ± 1.47	0.094
Hb (g/L)	130.58 ± 23.53	127.31 ± 17.96	118.86 ± 19.39**^##^	0.000
TBIL (μmol/L)	11.48 ± 3.85	12.29 ± 5.54	11.33 ± 4.36	0.263
DBIL (μmol/L)	4.11 ± 1.53	4.35 ± 2.22	4.20 ± 1.63	0.660
IBIL (μmol/L)	7.37 ± 2.67	7.93 ± 3.89	7.12 ± 3.25	0.175
ABI	1.04 ± 0.15	1.03 ± 0.13	0.96 ± 0.20*^#^	0.010
VPT (V)	16.50 ± 8.81	19.02 ± 10.15	20.98 ± 10.95*	0.014
OSTA	1.23 ± 0.35	−0.49 ± 0.23**	−2.60 ± 0.27**^##^	0.000
Bone metabolism				
LS BMD (g/cm^2^)	1.120 ± 0.148	0.990 ± 0.102**	0.811 ± 0.114**^##^	0.000
LS T score	0.40 ± 0.16	−0.75 ± 0.09**	−2.43 ± 0.10**^##^	0.000
FN BMD (g/cm^2^)	0.926 ± 0.107	0.805 ± 0.086**	0.678 ± 0.094**^##^	0.000
FN T score	−0.46 ± 0.11	−1.42 ± 0.07**	−2.18 ± 0.07**^##^	0.000
TH BMD (g/cm^2^)	0.875 ± 0.100	0.759 ± 0.081**	0.623 ± 0.085**^##^	0.000
TH T score	−0.85 ± 0.08	−1.64 ± 0.06**	−2.72 ± 0.06**^##^	0.000
Fragility fractures	0 (0.00%)	0 (0.00%)	25 (20.66%)* *^##^	0.000
Macrovascular complications				
Hypertension (n, %)	41 (56.16%)	69 (57.50%)	73 (60.33%)	0.830
CHD (n, %)	9 (12.33%)	20 (16.67%)	19 (15.70%)	0.710
Stroke (n, %)	10 (13.70%)	28 (23.33%)	36 (29.75%**)** *	0.039
PAD (n, %)	5 (6.85%)	13 (10.83%)	23 (19.01%) *	0.034
Microvascular complications				
DN (n, %)	27 (36.99%)	47 (39.17%)	63 (52.07%)	0.056
DR (n, %)	10 (13.70%)	17 (14.17%)	13 (10.74%)	0.701
DPN (n, %)	17 (23.29%)	38 (31.67%)	41 (33.88%)	0.285
Hypoglycemic drugs				
Metformin	43 (58.90%)	61 (50.83%)	66 (54.55%)	0.453
Sulfonylurea	27 (36.99%)	49 (40.83%)	53 (43.80%)	0.682
Alpha-glucosidase inhibitor	18 (24.66%)	42 (35.00%)	31 (25.62%)	0.165
Insulin	30 (41.10%)	64 (53.33%)	51 (42.15%)	0.168
Antihypertensive drugs				
ACEI	7 (9.59%)	14 (11.67%)	10 (8.26%)	0.690
ARB	23 (31.51%)	35 (29.17%)	38 (31.40%)	0.879
Beta-blockers	5 (6.85%)	11 (9.17%)	9 (7.44%)	0.837
α-blockers	4 (5.48%)	3 (2.50%)	5 (4.13%)	0.546
Calcium channel blockers	21 (28.77%)	29 (24.17%)	35 (28.93%)	0.619

Data are mean ± SD. *SD* standard deviation; *BMI* body mass index; *SBP* systolic blood pressure; *DBP* diastolic blood pressure; *PP* pulse pressure; *TC* total cholesterol; *TG* triglyceride; *HDL-C* high-density lipoprotein cholesterol; *LDL-C* low-density lipoprotein cholesterol; *FBG* fasting blood glucose; *HbA1c* glycated hemoglobin A1c; Calcium (mg/dL)^a^ = serum calcium concentration (mg/dL) + 0.8 × [4.0(g/dL)- serum albumin concentration (g/dl)]; *ALP* alkaline phosphatase; *eGFR* stimated glomerular filtration rate; *ACR* albumin- to-creatinine ratio; *WBC* white blood cell; *NLR* neutrophil to lymphocyte ratio; *Hb* hemoglobin; *TBIL* total bilirubin, *DBIL* direct bilirubin, *IBIL* indirect bilirubin, *ABI* Ankle-brachial index; *VPT* vibration perception threshold; *OSTA* osteoporosis self-assessment tool for Asians; *LS* lumbar spine; *FN* femoral neck; *TP* total hip; *CHD* coronary heart disease; *PAD* peripheral arterial disease; *DN* diabetic nephropathy; *DR* diabetic retinopathy; *DPN* diabetic peripheral neuropathy; *ACEI* angiotensin converting enzyme inhibitors; *ARB* angiotensin receptor antagonists; *T2DM* type 2 diabetes mellitus. Vs. Normal group, *P < 0.05, **P < 0.01; vs. Osteopenia group, ^#^*P* < 0.05, ^##^*P* < 0.01

### Association of circulating BNP with anthropometric, biochemical and clinical parameters in study subjects

In T2DM patients, circulating BNP levels had positive correlation with age, diabetic duration, cystatin C, NLR, and prevalence of osteoporosis, hypertension **(**SBP and PP), PAD, DR, DPN (VPT values), and DN (urinary ACR and creatinine), and negative correlation with TG, FBG, bilirubin (TBIL and IBIL), eGFR, calcium, lymphocyte count, Hb, OSTA, and BMD at different skeletal sites and corresponding T scores (*P* < 0.01 or *P* < 0.05; Table 2).

**Table 2:** Linear correlation analysis of variables associated with circulating BNP in study subjects

Variables	***r***	***P Value***	Variables	***r***	***P Value***
Gender	0.107	0.058	NLR	0.152	0.008
Diabetic duration	0.182	0.001	Fibrinogen	0.107	0.142
Age	0.345	0.000	Hb	−0.431	0.000
BMI	− 0.014	0.809	TBIL	−0.131	0.022
Height	−0.074	0.204	DBIL	−0.046	0.419
Weight	−0.047	0.417	IBIL	−0.164	0.004
SBP	0.271	0.000	ABI	−0.079	0.196
DBP	−0.095	0.091	VPT	0.190	0.002
PP	0.348	0.000	OSTA	−0.247	0.000
TC	−0.086	0.135	LS BMD	−0.154	0.006
TG	−0.158	0.006	LS T score	−0.157	0005
HDL-C	0.067	0.249	FN BMD	−0.287	0.000
LDL-C	−0.051	0.381	FN T score	−0.241	0.000
FBG	−0.126	0.026	TH BMD	−0.302	0.000
HbA1c	−0.083	0.141	TH T score	−0.306	0.000
Calcium	−0.217	0.000	Fragility fractures	0.055	0.336
ALP	0.047	0.408	Osteoporosis	0.239	0.000
Creatinine	0.113	0.046	Hypertension	0.150	0.008
eGFR	−0.246	0.000	CHD	0.094	0.098
ACR	0.359	0.000	Stroke	0.049	0.391
Cystatin C	0.253	0.000	PAD	0.212	0.000
WBC	−0.042	0.459	DN	0.281	0.000
Neutrophil count	0.017	0.762	DR	0.113	0.045
Lymphocyte count	−0.213	0.000	DPN	0.133	0.018

### Multivariable-adjusted ORs for the association of circulating BNP with increased presence of osteoporosis in study subjects

As shown in Table 3, univariate logistic regression analysis revealed that BMI, TG, HbA1c, calcium, lymphocyte count, and Hb were associated negatively with the presence of osteoporosis, whereas gender, age, diabetic duration, PP, HDL-C, NLR, circulating BNP, VPT, and prevalence of stroke, PAD, and DN positively correlated with the presence of osteoporosis. Importantly, circulating BNP remained independently significantly associated with the presence of osteoporosis after adjusting for all confounding variables when assessed in a multiple logistic regression model (OR, 2.710; 95% CI, 1.690–4.344; *P* < 0.01).

**Table 3:** Binary logistic regression analyses of variables contributing to osteoporosis in patients with T2DM

Variables	Univariate analysis			Multivariate analysis		
	B	OR (95% CI)	***P Value***	B	OR (95% CI)	***P Value***
BNP	0.997	2.710 (1.690–4.344)	0.000	1.549	4.706 (1.363–16.250)	0.014
Gender	2.019	7.528 (3.743–15.139)	0.000	3.382	29.419 (3.754–230.558)	0.001
Age	0.104	1.110 (1.071–1.151)	0.000	0.155	1.168 (1.073–1.271)	0.000
Diabetic duration	0.051	1.053 (1.006–1.102)	0.027			
BMI	−0.080	0. 923 (0.853–0.999)	0.046			
PP	0.018	1.018 (1.003–1.033)	0.018			
TC	−0.075	0.928 (0.721–1.194)	0.560			
TG	−0.256	0.774 (0.608–0.986)	0.038			
HDL-C	1.162	3.197 (1.208–8.460)	0.019			
LDL-C	−0.082	0.922 (0.677–1.254)	0.603			
FBG	−0.005	0.995 (0.941–1.051)	0.848			
HbA1c	−0.126	0.882 (0.782–0.995)	0.041			
Calcium	−0.266	0.766 (0.607–0.967)	0.025			
ALP	0.001	1.001 (0.994–1.007)	0.798			
Cystatin C	0.522	1.685 (0.923–3.075)	0.089			
WBC count	0.050	1.051 (0.956–1.157)	0.304			
Neutrophil count	0.092	1.097 (0.981–1.225)	0.103			
Lymphocyte count	−0.747	0.474 (0.283–0.794)	0.005			
NLR	0.145	1.156 (1.019–1.311)	0.024			
Fibrinogen	0.160	1.174 (0.879–1.568)	0.277			
Hb	−0.031	0.969 (0.952–0.986)	0.000			
TBIL	−0.009	0.991 (0.924–1.063)	0.804			
DBIL	0.036	1.037 (0.860–1.249)	0.704			
IBIL	−0.027	0.974 (0.884–1.072)	0.586			
VPT	0.048	1.049 (1.012–1.088)	0.009			
Hypertension	0.171	1.187 (0.659–2.138)	0.568			
CHD	0.281	1.325 (0.565–3.107)	0.518			
Stroke	0.901	2.462 (1.133–5.350)	0.023			
PAD	1.161	3.192 (1.156–8.811)	0.025			
DN	0.615	1.851 (1.022–3.352)	0.042			
DR	−0.277	0.758 (0.314–1.830)	0.538			

Beta is the standardized coefficient and measures the influence of each variable on osteoporosis; OR is the odds ratio and refers to the risk of osteoporosis

### Parameters related to diabetic osteoporosis across quartiles of circulating BNP levels in patients with T2DM

As shown in Table 4, the result indicated that BMD at the FN and TH and corresponding T scores were progressively decreased, and age and the prevalence of osteoporosis was progressively increased with increasing circulating BNP quartiles (all *P* for trend< 0.01; Table 4). Compared to the lowest quartile of BNP, the highest quartile had significantly older age and higher risk of prevalence of osteoporosis (all *P* < 0.01).

**Table 4:** Parameters related to diabetic osteoporosis across quartiles of circulating BNP levels in patients with T2DM

Variables		Circulating BNP quintiles			***P Value***
	Q1 (*n* = 78)	Q2 (*n* = 79)	Q3 (*n* = 79)	Q4 (*n* = 78)	
Age (years)	59.59 ± 9.10	64.04 ± 9.91**	68.11 ± 8.89**^##^	68.53 ± 9.89**^##^	0.000
LS BMD (g/cm^2^)	0.998 ± 0.154	0.995 ± 0.189	0.968 ± 0.183	0.942 ± 0.173*	0.151
LS T score	− 0.922 ± 0.155	−0.959 ± 0.189	−1.204 ± 0.184	−1.434 ± 0.175	0.144
FN BMD (g/cm^2^)	0.844 ± 0.129	0.797 ± 0.139 *	0.764 ± 0.125**	0.733 ± 0.121**^##^	0.000
FN T score	−1.075 ± 0.120	−1.415 ± 0.124 *	− 1.599 ± 0.106 **	−1.881 ± 0.110 **^##^	0.000
TH BMD (g/cm^2^)	0.787 ± 0.124	0.755 ± 0.136	0.713 ± 0.127 **^#^	0.680 ± 0.113 **^##^	0.000
TH T score	−1.460 ± 0.103	−1.718 ± 0.113	−2.039 ± 0.110** ^##^	−2.274 ± 0.098**^##^	0.000
Fragility fractures (n, %)	5 (6.41%)	7 (8.86%)	4 (5.06%)	9 (11.54%)	0.359
Osteoporosis (n, %)	18 (23.08%)	28 (35.44%)	36 (45.57%)*	39 (50.00%)* *	0.003
Osteopenia (n, %)	31 (39.74%)	34 (43.04%)	30 (37.97%)	25 (32.05%)	0.551

Data are mean ± SD. vs. Q1, ^*^P < 0.05, ^**^P < 0.01; vs. Q2, ^#^*P* < 0.05,^##^*P*<0.01

### The predictive value of circulating BNP in detecting osteoporosis in patients with T2DM

**Fig. 2:**
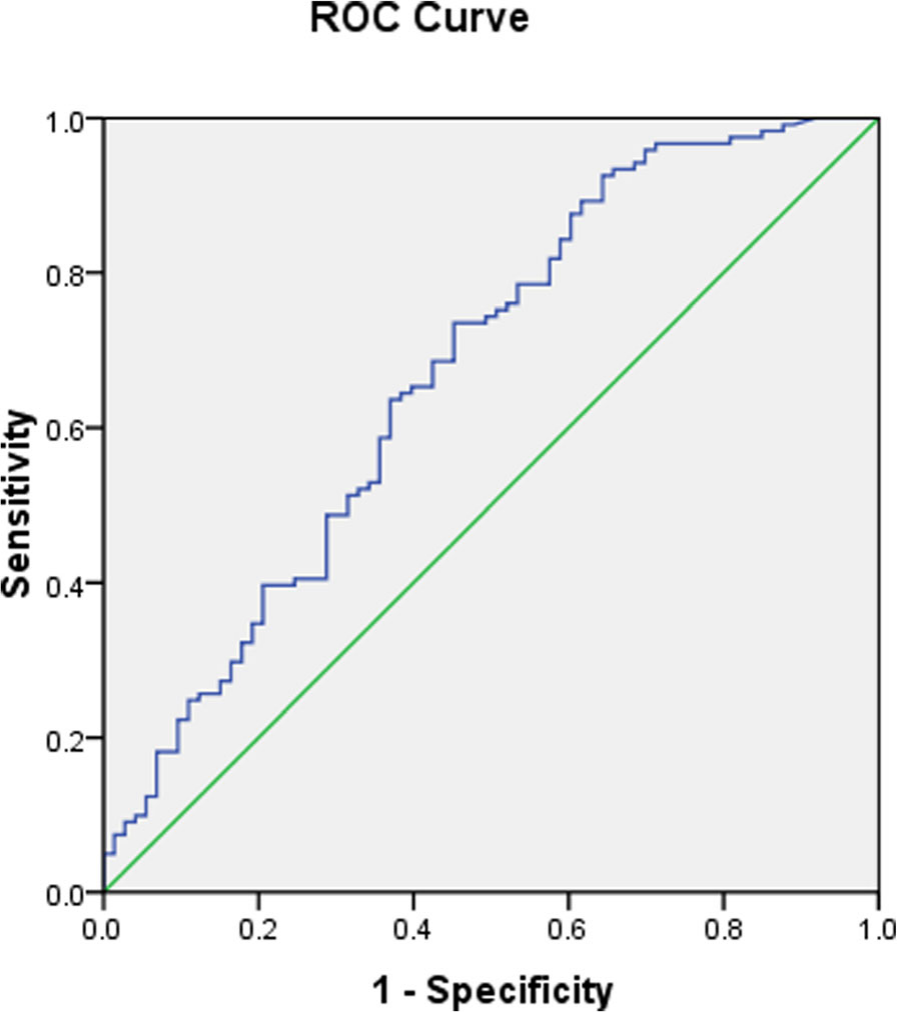
ROC analysis of circulating B-type natriuretic peptide (BNP) to indicate osteoporosis for T2DM patients. AUC = 0.671; 95% CI, 0.591–0.752; *P* = 0.000; identified BNP cutoff value = 16.35 pg/ml; Youden index = 0.284; sensitivity: 73.6%; specificity: 54.8%

The results of ROC curves revealed that the best cutoff value for circulating BNP to predict osteoporosis was 16.35 pg/ml (sensitivity: 73.6%, specificity: 54.8%, and area under curve 0.671) in patients with T2DM (Fig.2).

## Discussion

To date, this was the first study to explore the relationship between circulating BNP and the risk of diabetic osteoporosis. We found that circulating BNP significantly increased in T2DM patients with osteoporosis, and was independently and positively correlated with the prevalence of osteoporosis. Moreover, BMD at the FN and TH and corresponding T scores were progressively decreased, whereas the prevalence of osteoporosis was progressively increased with increasing circulating BNP quartiles. Further, we showed that circulating BNP was found to predict the presence of diabetic osteoporosis. These findings suggest that circulating BNP may be a useful biomarker of osteoporosis, and high circulating BNP may associate with an increased risk of diabetic osteoporosis.

BNP, a 32-amino acid neurohormone, and its inactive cleavage product NT-proBNP are predominantly synthesized at equimolar levels by the ventricle myocytes and release into the circulation in response to ventricular dilatation, pressure overload or myocardial ischemia [13, 14]. Wang et al. and Lee et al. found that increased serum NT-proBNP was associated with lower T-scores at the LS, and serum NT-proBNP was independently and negatively associated with poor BMD values at the LS in peritoneal dialysis patients and renal transplant recipients [16, 17]. Moreover, Kajita et al. performed a quantitative trait locus analysis and a longitudinal follow-up study of 378 Japanese postmenopausal women over 5 years, and showed that genetic variation of BNP gene may be an important determinant of postmenopausal osteoporosis, in part through the mechanism of accelerated bone loss [24]. In agreement with previous studies, we found that T2DM patients with osteoporosis had significantly higher circulating BNP, and circulating BNP was correlated positively with the prevalence of osteoporosis, and negatively with BMD at different skeletal sites and corresponding T scores, and OSTA, which could be used conservatively to identify individuals who are likely to have low BMD and classify the risk of osteoporosis [21]. Moreover, circulating BNP remained independently significantly associated with the presence of osteoporosis after adjusting for all confounding variables. Additionally, circulating BNP was found to predict the presence of diabetic osteoporosis. These data suggest that altered circulating BNP and genetic variation of BNP may play an important role in the development of diabetic osteoporosis. Considering that the potential osteoprotective property of BNP reported by some studies [7, 9, 13–17] and the cumulative effects of natriuretic peptides oppose the physiologic abnormalities of HF, instead of begetting HF [7], there is a possibility that circulating BNP may be upregulated to compensate for lower BMD and increased risk of osteoporosis in response to cardio-metabolic risk factors, inflammation orvascular damage in T2DM patients, and circulating BNP might be a potential biomarker for osteoporosis in patients with T2DM. However, further investigations are needed to confirm the potential role of circulating BNP in the development of diabetic osteoporosis.

Numerous studies have demonstrated that chronic low-grade inflammation and oxidative stress were involved in the initiation and development of osteoporosis [3, 22, 25, 26]. NLR has been reported as a novel marker for systemic inflammation in recent studies [27]. There is evidence that bilirubin at normal to mildly elevated levels, a byproduct of normal hemoglobin breakdown in mammals, exerts anti-oxidative, anti-inflammatory, and immunoregulatory activities [19, 20]. Our study showed that T2DM patients with osteoporosis had significantly higher NLR and nonsignificantly increased fibrinogen, and significantly lower lymphocyte counts and Hb and nonsignificantly decreased bilirubin. Moreover, lymphocyte counts, NLR, and Hb were associated with the presence of diabetic osteoporosis. These findings provided further evidence that a dysregulated inflammatory response played an important role in the development of diabetic osteoporosis. Exposure of cultured rat myocytes to lipopolysaccharides and pro-inflammatory cytokines was associated with a increase in expression of BNP mRNA and secretion of protein in a dose-dependent manner [28]. Similar results were obtained in cultured neonatal rat cardiocytes and rat ventricular myocytes [29]. Additionally, in vitro in human adipocytes, BNP enhances adiponectin secretion, which has anti-inflammatory properties [30], indirectly suggesting that BNP may exert anti-inflammatory effect. Clinical studies also demonstrated that inflammation might lead to increased secretion or decreased degradation of natriuretic peptide, which might explain, at least partly, elevated levels of natriuretic peptide [29]. These findings together revealed that BNP may exert an anti-inflammatory effect, and circulating BNP may compensatorily increase in response to inflammatory. Consistent with previous studies [31], our data showed that circulating BNP was positively associated with NLR, and negatively with lymphocyte count, bilirubin, and Hb. Collectively, these data suggested that a compensatory increase in circulating BNP due to inflammation may exert beneficial effects on bone metabolism in patients with diabetic osteoporosis, further studies are needed to fully elucidate its mechanism of action.

There is growing evidence that systemic vasculopathy due to increased inflammation and oxidative stress may lead to atherosclerosis-related intraosseous ischemia and may be involved in the development of osteoporosis and fractures [3, 22, 32]. ABI, a noninvasive diagnostic biomarker for lower-extremity PAD, has been reported to be associated with increased incidence of cardiovascular events and mortality. VPT, an indicator of confirmed clinical neuropathy, can reflect the clinical severity of DPN. Our present results provide further evidence that diabetic microvascular and macrovascular complications may contribute to the development of diabetic osteoporosis by decreasing blood flow to the bones and influencing bone turnover, since we showed that T2DM patients with osteoporosis had significantly lower eGFR, higher VPT values, arterial stiffness as measured by PP, and prevalence of stroke and PAD as reflected by lower ABI compared with individuals with normal BMD, and, moreover univariate logistic regression analysis revealed that the prevalence of stroke, PAD, PP, DN, and VPT values were associated with the presence of osteoporosis, in accordance with previous studies [3, 22, 32, 33]. We further showed that circulating BNP had significant positive correlation with PP and prevalence of PAD, DR, DPN as evidenced by higher VPT, and DN as reflected by elevated serum creatinine, urinary ACR, and cystatin C, and lower eGFR, in line with previous reports [34–41]. Chronic excess of BNP in mice was reported to prevent diabetic glomerular injury, and improve albuminuria and renal dysfunction [35]. Welsh et al. performed a case-cohort study of 439 patients with incident microvascular events (new or worsening DN or DR) and 2946 noncase subjects, and found that plasma NT-proBNP was higher among case subjects, and the hazard ratios for microvascular events per 1-SD increase in NT- proBNP was 1.63 after adjustment for potential confounding factors [36]. Two studies conducted by Jurado et al. and Hamano et al. have reported a significant positive relationship between NT-proBNP levels and the presence of DPN, independently of previous cardiovascular disease and related risk factors in T2DM patients [34, 36]. Transgenic mice with overexpression of BNP have accelerated vascular regeneration in response to hind limb ischemia after experimental femoral artery ligation [37], and higher levels of circulating NT-proBNP have rencently been reported in patients with PAD [34, 38]. These data together would indicate the possibility that ischemia and hypoxia due to diabetic vascular diseases can lead to BNP production via wall motion abnormalities and increased wall stress or that ischemia can directly promote BNP to release, independent of cardiomyocytes [39, 40], and compensatory increase in circulating BNP in patients with diabetic osteoporosis may exert osteoprotective effect by promoting vessel creation, regulating vascular inflammation, improving endothelial function, atherosclerosis and blood supply to the bones [38]. However, the protective effect of BNP may be weakened due to BNP receptors impairment in atherosclerosis or ischemic vascular disease [41], but the detailed mechanism is still unrevealed.

Growing evidence suggests that cardiometabolic risk factors, including female subjects, advanced age, lower weight and BMI, longer diabetic duration, dyslipidemia, hypertension, and hyperglycemia, play an important role in the pathogenesis of diabetic osteoporosis [9, 16, 17, 22, 32]. Our study findings provided further evidence that patients with diabetic osteoporosis had significantly more female subjects, older age and lower weight, and nonsignificantly longer diabetic duration, higher SBP and lower BMI, and, further, gender, age, BMI, diabetic duration, HbA1c, TG, and HDL-C were associated with the presence of osteoporosis. Moreover, circulating BNP correlated significantly and positively with age, diabetic duration, SBP, and prevalence of hypertension, and correlated negatively with FBG and TG, in agreement with previous studies [15, 40, 42–44]. Grauslund and colleagues also reported a positive association of NT-proBNP with age and duration in T1DM patients, and women patients had an increase in NT-proBNP due to the fact that estrogens induce BNP production [15, 43].

Prior studies have demonstrated that circulating NT-proBNP was elevated in patients with hypertension, and plasma NT-pro BNP was positively related to blood pressure levels, especially SBP [40, 44]. Clinical studies showed that natriuretic peptides had the properties of dissolving and regulating lipid, and promoting lipid oxidation [7, 15, 16, 40], and cardiac TG content rose due to downregulation of cardiac BNP expression in animal models of obesity [40]. Epidemiological and prospective studies have shown that circulating NT-proBNP was negatively associated with FBG [42] and was a negative predictor of new-onset T2DM [15]. Recent studies have shown that short-term and chronic BNP infusion improves glucose tolerance and blood glucose control in healthy volunteers and in obese diabetic db/db mice, respectively [15]. Both our hereby presented findings and results of previous studies suggest that circulating BNP was associated with cardio-metabolic risk factors, and may play an important role in the development of diabetic osteoporosis via antidiabetic, lipids-lowering, and BP-lowering effects, further studies are needed to fully elucidate its mechanism of action.

The present study has several limitations. First, the cross-sectional design of the study did not permit us to determine any causal relationships of circulating BNP with diabetic osteoporosis. Thus, further large-scale longitudinal studies are needed to comfirm our results. Second, imaging examination was not performed in all T2DM patients. Thus, highly prevalent non-symptomatic vertebral fractures cannot be ruled out, which would likely underestimate the rule prevalence of diabetic osteoporosis and fractures. Remarkably, though, self-report of previous physician diagnosis is often used to assess the rate of osteoporosis, fractures, and/or diabetes in large-scale population studies. We were also unable to distinguish between different types of fractures, given the small number of people for each type of fracture. Third, we did not evaluate the family history, physical activity, dietary habits, falls, and socioeconomic status that are risk factors of diabetic osteoporosis, and measure bone metabolism markers (e.g., vitamin D and parathyroid hormone, tartrate-resistant acid phosphatase 5b, type I procollagen amino terminal peptide, and C-terminal cross-linking type I collagen of type I collagen). Despite these limitations, the current study has some advantages, including relatively large sample size, use of a standardized method at a single center, and thoroughly adjustment for possible confounders, which can raise the reliability of our findings. Moreover, our study, to our knowledge, provides first clinical evidence regarding the link between circulating BNP and diabetic osteoporosis.

## Conclusions

The present study showed that circulating BNP significantly increased in T2DM patients with osteoporosis, and circulating BNP was independently and positively correlated with the prevalence of diabetic osteoporosis, thereby suggesting that circulating BNP may be used as a useful biomarker of risk of osteoporosis in T2DM patients. However, further prospective, large-scale, randomized controlled studies are warranted to establish our results, and elucidate the underlying mechanism of the association.

## Data Availability

The datasets generated during and analyzed during the current study are available from the corresponding author on reasonable request.

## Abbreviations

BNP: B-type natriuretic peptide
BMD: Bone mineral density
T2DM: Type 2 diabetes mellitus
HF: Heart failure
RAAS: Renin-angiotensin-aldosterone system
NYHA: New York Heart Association
RAS: Renin-angiotensin system
NT-proBNP: N-terminal pro-BNP
LS: Lumbar spine
DR: Diabetic retinopathy
DN: Diabetic nephropathy
DPN: Diabetic peripheral neuropathy
PAD: Peripheral arterial disease
CHD: Coronary heart disease
BMI: Body mass index
SBP: Systolic blood pressure
DBP: Diastolic blood pressure
PP: Pulse pressure
FBG: Fasting blood glucose
HbA1c: Glycated hemoglobin A1c
TC: Total cholesterol
TG: Triglyceride
HDL-C: High-density lipoprotein cholesterol
LDL-C: Low-density lipoprotein cholesterol
TBIL: Total bilirubin
DBIL: Direct bilirubin
IBIL: Indirect bilirubin
ALP: Alkaline phosphatase
Hb: Hemoglobin
WBC: White blood cell
NLR: Neutrophil to lymphocyte ratio
ACR: Albumin-to-creatinine ratio
eGFR: estimated glomerular filtration rate
ABI: Ankle–brachial index
VPT: Vibration perception threshold
SWM: Semmes-Weinstein monofilament
OSTA: Osteoporosis Self-Assessment Tool for Asians
FN: Femoral neck
TH: Total hip
DXA: Dual X-ray absorptiometry
SD: Standard deviation
ANOVA: One-way analysis of variance
OR: Odds ratios
CI: Confidence intervals
ROC: Receiver operating characteristic

## References

[1] Li Y, Zhao Z, Wang L, Fu Z, Ji L, Wu X (2020). The Prevalence of Osteoporosis Tested by Quantitative Computed Tomography in Patients With Different Glucose Tolerances. J Clin Endocrinol Metab.

[2] Sanches CP, Vianna AGD, Barreto FC (2017). The impact of type 2 diabetes on bone metabolism. Diabetol Metab Syndr.

[3] Paschou SA, Anagnostis P, Vryonidou A, Goulis DG (2018). Diabetes and atherosclerosis: old players in a new field, Osteoporosis. Curr Vasc Pharmacol.

[4] Mohsin S, Kaimala S, Sunny JJ, Adeghate E, Brown EM (2019). Type 2 diabetes mellitus increases the risk to hip fracture in postmenopausal osteoporosis by deteriorating the trabecular bone microarchitecture and bone mass. J Diabetes Res.

[5] Suzuki K, Sugimoto C, Takizawa M, Ishizuka S, Kikuyama M, Togawa H (2000). Correlations between bone mineral density and circulating bone metabolic markers in diabetic patients. Diabetes Res Clin Pract.

[6] Ueland T, Dahl CP, Kjekshus J, Hulthe J, Böhm M, Mach F (2011). Osteoprotegerin predicts progression of chronic heart failure: results from CORONA. Circ Heart Fail.

[7] Chen YH, Wu YW, Yang WS, Wang SS, Lee CM, Chou NK (2012). Relationship between bone mineral density and serum osteoprotegerin in patients with chronic heart failure. PLoS One.

[8] Verheyen N, Fahrleitner-Pammer A, Belyavskiy E, Gruebler MR, Dimai HP, Amrein K (2017). Relationship between bone turnover and left ventricular function in primary hyperparathyroidism: the EPATH trial. PLoS One.

[9] Szekanecz Z, Raterman HG, Pethő Z, Lems WF (2019). Common mechanisms and holistic care in atherosclerosis and osteoporosis. Arthritis Res Ther.

[10] Shiga T, Hosaka F, Wakaumi M, Matsuda N, Tanizaki K, Kajimoto K (2003). Amiodarone decreases plasma brain natriuretic peptide level in patients with heart failure and ventricular tachyarrhythmia. Cardiovasc Drugs Ther.

[11] Balion CM, Santaguida P, McKelvie R, Hill SA, McQueen MJ, Worster A (2008). Physiological, pathological, pharmacological, biochemical and hematological factors affecting BNP and NT-proBNP. Clin Biochem.

[12] Lin YS, Chu PH, Kuo MC, Jung SM, Lim KE, Kuo CT (2006). Use of a B-type natriuretic peptide in evaluating the treatment response of a relapsed lymphoma with cardiac involvement. Int J Hematol.

[13] Beer S, Golay S, Bardy D, Feihl F, Gaillard RC, Bachmann C (2005). Increased plasma levels of N-terminal brain natriuretic peptide (NT-proBNP) in type 2 diabetic patients with vascular complications. Diabetes Metab.

[14] Cao Z, Jia Y, Zhu B (2019). BNP and NT-proBNP as diagnostic biomarkers for cardiac dysfunction in both clinical and forensic medicine. Int J Mol Sci.

[15] Moro C (2016). Targeting cardiac natriuretic peptides in the therapy of diabetes and obesity. Expert Opin Ther Targets.

[16] Wang CH, Tsai JP, Lai YH, Lin YL, Kuo CH, Hsu BG (2016). Inverse relationship of bone mineral density and serum level of N-terminal pro-B-type natriuretic peptide in peritoneal dialysis patients. Ci Ji Yi Xue Za Zhi.

[17] Lee MC, Lee CJ, Shih MH, Ho GJ, Chen YC, Hsu BG (2014). N-terminal pro-B-type natriuretic peptide is inversely related to bone mineral density in renal transplant recipients. Transplant Proc.

[18] Suda M, Ogawa Y, Tanaka K, Tamura N, Yasoda A, Takigawa T (1998). Skeletal overgrowth in transgenic mice that overexpress brain natriuretic peptide. Proc NatlAcad Sci U S A.

[19] Yan P, Zhang Z, Wan Q, Zhu J, Li H, Gao C (2018). Association of serum uric acid with bone mineral density and clinical fractures in Chinese type 2 diabetes mellitus patients: a cross-sectional study. Clin Chim Acta.

[20] Yan P, Zhang Z, Miao Y, Xu Y, Zhu J, Wan Q (2019). Physiological serum total bilirubin concentrations were inversely associatedwith diabetic peripheral neuropathy in Chinese patients with type 2 diabetes: a cross-sectional study. Diabetol Metab Syndr.

[21] Wang P, Abdin E, Shafie S, Chong SA, Vaingankar JA, Subramaniam M (2019). Estimation of prevalence of osteoporosis using OSTA and its correlation with Sociodemographic factors, disability and comorbidities. Int J Environ Res Public Health.

[22] Pinheiro MM, Ciconelli RM, Martini LA, Ferraz MB (2009). Clinical risk factors for osteoporotic fractures in Brazilian women and men: the Brazilian osteoporosis study (BRAZOS). Osteoporos Int.

[23] Huang N, Zhou J, Wang W, Wang Q, Tang Y, Sun Y (2018). Retinol-binding protein 4 is positively associated with bone mineral density in patients with type 2 diabetes and osteopenia or osteoporosis. Clin Endocrinol.

[24] Kajita M, Ezura Y, Iwasaki H, Ishida R, Yoshida H, Kodaira M (2003). Association of the -381T/C promoter variation of the brain natriuretic peptide gene with low bone-mineral density and rapid postmenopausal bone loss. J Hum Genet.

[25] Buizert PJ, van Schoor NM, Lips P, Deeg DJ, Eekhoff EM (2009). Lipid levels: a link between cardiovascular disease and osteoporosis?. J Bone Miner Res.

[26] An Y, Zhang H, Wang C, Jiao F, Xu H, Wang X (2019). Activation of ROS/MAPKs/NF-κB/NLRP3 and inhibition of efferocytosis in osteoclast-mediated diabetic osteoporosis. FASEB J.

[27] Del Turco S, Basta G, De Caterina AR, Sbrana S, Paradossi U, Taddei A (2019). Different inflammatory profile in young and elderly STEMI patients undergoing primary percutaneouscoronary intervention (PPCI): its influence on no-reflow and mortality. Int J Cardiol.

[28] Rudiger A, Fischler M, Harpes P, Gasser S, Hornemann T, von Eckardstein A (2008). In critically ill patients, B-type natriuretic peptide (BNP) and N-terminal pro-BNP levels correlate with C-reactive protein values and leukocyte counts. Int J Cardiol.

[29] Rudiger A, Gasser S, Fischler M, Hornemann T, von Eckardstein A, Maggiorini M (2006). Comparable increase of B-type natriuretic peptide and amino-terminal pro- B-type natriuretic peptide levels in patients with severe sepsis, septic shock, and acute heart failure. Crit Care Med.

[30] Liu Y, Vu V, Sweeney G (2019). Examining the Potential of Developing and Implementing Use of Adiponectin-Targeted Therapeutics for Metabolic and Cardiovascular Diseases. Front Endocrinol (Lausanne).

[31] Solomon SD, Anavekar N, Skali H, McMurray JJ, Swedberg K (2005). Influence of ejection fraction on cardiovascular outcomes in a broad spectrum of heart failure patients. Circulation..

[32] Laroche M, Pécourneau V, Blain H, Breuil V, Chapurlat R, Cortet B (2017). Osteoporosis and ischemic cardiovascular disease. Joint Bone Spine.

[33] Starup-Linde J, Vestergaard P (2015). Management of endocrine disease: diabetes and osteoporosis: cause for concern?. Eur J Endocrinol.

[34] Hamano K, Nakadaira I, Suzuki J, Gonai M (2014). N-terminal fragment of probrain natriuretic peptide is associated with diabetes microvascular complications in type 2 diabetes. Vasc Health Risk Manag.

[35] Makino H, Mukoyama M, Mori K, Suganami T, Kasahara M, Yahata K (2006). Transgenic overexpression of brain natriuretic peptide prevents the progression of diabetic nephropathy in mice. Diabetologia..

[36] Jurado J, Ybarra J, Ferrandiz M, Comerma L, Pou JM (2007). Amino-terminal brain natriuretic peptide is related to the presence of diabetic polyneuropathy independently of cardiovascular disease. Diabetes Care.

[37] Yamahara K, Itoh H, Chun TH, Ogawa Y, Yamashita J, Sawada N (2003). Significance and therapeutic potential of the natriuretic peptides/cGMP/cGMP-dependent protein kinase pathway in vascular regeneration. Proc Natl Acad Sci U S A.

[38] Fan J, Jouni H, Khaleghi M, Bailey KR, Kullo IJ (2012). Serum N-terminal pro-B-type natriuretic peptide levels are associated with functional capacity in patients with peripheral arterial disease. Angiology.

[39] Welsh P, Woodward M, Hillis GS, Li Q, Marre M, Williams B (2014). Do cardiac biomarkers NT-proBNP and hsTnT predict microvascular events in patients with type 2 diabetes? Results from the ADVANCE trial. Diabetes Care.

[40] Gruden G, Landi A, Bruno G (2014). Natriuretic peptides, heart, and adipose tissue: new findings and future developments for diabetes research. Diabetes Care.

[41] Jin QH, Ye WL, Chen HH, He XJ, Li TL, Liu Q (2014). Levels of brain natriuretic peptide are associated with peripheral arterial disease in subjects with type-2 diabetes mellitus. BMC Endocr Disord.

[42] Sung SH, Chuang SY, Sheu WH, Lee WJ, Chou P, Chen CH (2009). Relation of adiponectin and high-sensitivity C-reactive protein to pulse-wave velocity and N-terminal pro-B-type natriuretic peptide in the general population. Am J Cardiol.

[43] Bower JK, Lazo M, Matsushita K, Rubin J, Hoogeveen RC, Ballantyne CM (2015). N-terminal pro-brain natriuretic peptide (NT-proBNP) and risk of hypertension in the atherosclerosis risk in communities (ARIC) study. Am JHypertens.

[44] Sarzani R, Salvi F, Dessì-Fulgheri P, Rappelli A (2008). Renin-angiotensin system, natriuretic peptides, obesity, metabolic syndrome, and hypertension: an integrated view in humans. J Hypertens.

